# Semiphysiological versus Empirical Modelling of the Population Pharmacokinetics of Free and Total Cefazolin during Pregnancy

**DOI:** 10.1155/2014/897216

**Published:** 2014-02-03

**Authors:** J. G. Coen van Hasselt, Karel Allegaert, Kristel van Calsteren, Jos H. Beijnen, Jan H. M. Schellens, Alwin D. R. Huitema

**Affiliations:** ^1^Department of Clinical Pharmacology, Netherlands Cancer Institute, Plesmanlaan 121, P.O. Box 1066 CX, Amsterdam, The Netherlands; ^2^Department of Pharmacy & Pharmacology, Netherlands Cancer Institute/Slotervaart Hospital, Louwesweg 6, P.O. Box 90440, 1006 BK Amsterdam, The Netherlands; ^3^Department of Development and Regeneration, KU Leuven, Herestraat 49, 300 Leuven, Belgium; ^4^Neonatal Intensive Care Unit, University Hospitals Leuven, Herestraat 49, 300 Leuven, Belgium; ^5^Obstetrics and Gynecology, University Hospitals Leuven, Herestraat 49, 300 Leuven, Belgium; ^6^Division of Pharmacoepidemiology & Clinical Pharmacology, Department of Pharmaceutical Sciences, Faculty of Science, Utrecht University, P.O. Box 80125, 3508 TC Utrecht, The Netherlands

## Abstract

This work describes a first population pharmacokinetic (PK) model for free and total cefazolin during pregnancy, which can be used for dose regimen optimization. Secondly, analysis of PK studies in pregnant patients is challenging due to study design limitations. We therefore developed a semiphysiological modeling approach, which leveraged gestation-induced changes in creatinine clearance (CrCL) into a population PK model. This model was then compared to the conventional empirical covariate model. First, a base two-compartmental PK model with a linear protein binding was developed. The empirical covariate model for gestational changes consisted of a linear relationship between CL and gestational age. The semiphysiological model was based on the base population PK model and a separately developed mixed-effect model for gestation-induced change in CrCL. Estimates for baseline clearance (CL) were 0.119 L/min (RSE 58%) and 0.142 L/min (RSE 44%) for the empirical and semiphysiological models, respectively. Both models described the available PK data comparably well. However, as the semiphysiological model was based on prior knowledge of gestation-induced changes in renal function, this model may have improved predictive performance. This work demonstrates how a hybrid semiphysiological population PK approach may be of relevance in order to derive more informative inferences.

## 1. Introduction

Cefazolin is a cephalosporin antibiotic which is highly bound to serum albumin (75–85%) [1] but with substantial interindividual variability (IIV) [2, 3]. Cefazolin is effective against Gram-positive bacteria and is used as prophylaxis during surgical interventions [4] which may also be required during pregnancy or delivery [5]. Characterization of the pharmacokinetics (PK) of free (unbound) cefazolin and the associated IIV is of clinical relevance to further optimize dosing regimens, firstly because the free drug concentration is ultimately responsible for the pharmacological effect, and secondly because the free cefazolin plasma concentration has been shown to correlate better with the effect-site tissue concentrations compared to the total cefazolin plasma concentration [6]. However, a population PK model for free cefazolin, which could be used to rationally derive efficacious dose regimens for various infections, is not yet available.

During pregnancy, PK can be altered due to various gestation-induced physiological changes [7] and therefore can potentially require the conduct of PK studies in pregnant patients. However, frequently, the conduct of such studies is associated with practical limitations such as difficulties in obtaining blood samples at different occasions during pregnancy and the recruitment of sufficient numbers of eligible patients. As a result, the quantification of time-varying changes in PK based on such potentially subinformative studies can be challenging [8]. Yet this challenge emphasizes the need for analysis strategies that improve the quality of inferences derived from such studies.

In this analysis we aimed to address the aforementioned two challenges. The need for adequate characterization of unbound cefazolin PK was addressed by development of an empirical population PK model for both free and total cefazolin during pregnancy. Gestation-induced changes on PK parameters were also investigated.

Since cefazolin is mainly excreted in unchanged form via glomerular filtration and because the GFR increases during pregnancy, it is expected that the PK of cefazolin will change during pregnancy accordingly. The second objective of this work was to propose a new hybrid approach in which previously established time-varying and gestation-induced changes in the glomerular filtration rate (GFR) were leveraged into a population PK model for cefazolin, which may lead to a model with increased predictive value compared to an empirical model based only on the actual PK dataset.

## 2. Methods

### 2.1. Study Data

The analysis is based on one prospective clinical study and two previously published studies that contained individual-level PK data [3, 9].

In the prospective clinical study, cefazolin plasma observations (*n* = 153) were collected from pregnant women (*n* = 41) during a variety of *in utero* surgical interventions. The median gestational age (GA) at intervention was 25 (range 17–34) weeks. In 84% of cefazolin observations, free cefazolin concentrations were simultaneously available. Patients received intravenously (i.v.) administered cefazolin (2 g every 8 hours for 2 days), which is part of the routine clinical care for scheduled *in utero* surgeries. This study was approved by the ethical board of the University Hospitals Leuven, Belgium. Patients were included after providing written informed consent. Further details of the study including bioanalytical methods have been described elsewhere [3, 9].

The first literature study included in this analysis was based on a report by Fiore Mitchell et al. investigating cefazolin PK in term pregnancies at caesarean delivery [10]. Patients received 1 g cefazolin (i.v. bolus) shortly before elective cesarean. Plasma samples (*n* = 24) were collected at a mean time of 1.85 h after dose. A fixed GA of 40 gestational weeks was assumed for all patients in this study.

The second literature study included observations collected during 10 fetal interventions (*in utero* survival treatment of the fetus) in 7 women as described by Brown et al. [11], where mothers with a mean GA of 27 weeks received a single cefazolin dose of 2 g i.v. bolus, with a mean plasma sampling time of 0.5 h after dose.

Throughout the analysis we computed creatinine clearance with the Cockroft-Gault equation [12] as metric for glomerular filtration rate (GFR) using body weight as weight descriptor. A summary of patients demographics and dataset characteristics of the pooled dataset is depicted in Table 1.

### 2.2. Base Pharmacokinetic Model

A base population pharmacokinetic model was developed which was subsequently used as a starting point for the empirical and semiphysiological model development.

Mono-, bi-, and triexponential models with first-order and nonlinear elimination were considered. Nonlinear and linear protein binding models were considered for describing the relationship between free and total cefazolin plasma concentrations. Inclusion of IIV was considered for all structural model parameters as follows:

(1) $$\begin{array}{c} P_{i}=P\cdot \exp\left(\eta_{i}\right), \end{array}$$

where *P*_*i*_ is the individual parameter estimate for individual *i*, *P* is the typical population parameter estimate, and *η*_*i*_ was assumed to be distributed *N*(0, *ω* ^2^). Residual unexplained variability was implemented as either a proportional or combined error model:

(2) $$\begin{array}{c} C_{\text{observed},ij}=C_{\text{pred},ij}\times \left(1+\varepsilon_{p,ij}\right)+\varepsilon_{a,ij}, \end{array}$$

where *C*_total,*ij*_ represents the observed concentration for individual *i* and observation *j*, *C*_pred,*ij*_ represents the individual predicted concentration, and *ε*_*p*,*ij*_ and *ε*_*a*,*ij*_ represent the proportional and additive errors distributed following *N*(0, *σ* ^2^).

### 2.3. Empirical Gestational Effect Model

We considered the following covariate relationships: gestational age (weeks) related to clearance (CL), central volume, peripheral volume, and the free fraction of cefazolin. The rationale for evaluating distribution volume relationships was related to potential changes in body composition. We evaluated the relationship between the free fraction parameter because of potential changes in protein binding. Furthermore we evaluated the relationship between CL and creatinine clearance (CrCL). We considered both linear and power relationships:

(3) $$\begin{array}{c} P=\theta_{P0}+\theta_{P\text{preg}}\times \left(1+\left(\frac{\text{COV}}{N\text{COV}}\right)\right),\\ P=\theta_{P0}\times {\left(\frac{\text{COV}}{N\text{COV}}\right)}^{\theta_{P\text{preg}}}, \end{array}$$

where *P* represents the covariate-adjusted population parameter, *θ*_*P*0_ represents the baseline population parameter estimate for parameter *P*, *θ*_*P*preg_ represents the gestational covariate effect estimate for parameter *P*, and *N*COV represents a normalization factor for each covariate. For linear GA models this was the maximum GA of 40, for CrCL this was set to 125 mL/min, and for albumin this was set to 30 g/L.

### 2.4. Semiphysiological Gestational Effect Model

The semiphysiological model consisted of two parts. First a mixed effect model was developed to describe the individual predicted changes in CrCL based on the previously described mean change in CrCL and the individual observed CrCL values, as a surrogate for the change in GFR during pregnancy. Then, the mixed effect CrCL model was associated with the cefazolin clearance in the base pharmacokinetic model, as cefazolin is primarily renally eliminated.

#### 2.4.1. Creatinine Clearance Mixed Effect Model

The typical change in CrCL during pregnancy as described previously [13] was parameterized as follows:

(4) $$\begin{array}{c} \text{CrCL}\left(t\right)=\text{CrC}{\text{L}}_{0}+\frac{\text{CrC}{\text{L}}_{\text{MAX}}\times t}{\text{CrC}{\text{L}}_{50}+t}, \end{array}$$

where CrCL_0_ represented baseline (nonpregnant CrCL), CrCL_MAX_ represented maximum typical increase in CrCL, and CrCL_50_ represented the time of half-maximum change in CrCL. The typical parameter estimates for these three parameters were fixed to the previously estimated values (Table 3), which were based on a meta-analysis of the literature reporting gestational changes in CrCL [13]. Briefly, this was derived using a sample-size weighted regression analysis of two studies reporting the dynamics of CrCL during pregnancy [14, 15]. Random effects for IIV were considered for all three parameters. Residual variability was described using a proportional error model for individual *i* and observation *j* as follows:

(5) $$\begin{array}{c} {\text{CrCL}}_{\text{obs},ij}\left(t\right)={\text{CrCL}}_{\text{pred},ij}\left(t\right)\times \left(1+\varepsilon_{\text{CRCL},ij}\right), \end{array}$$

where CrCL_*ij*_ represents the observed CrCL value, CrCL_*i*_ represents the predicted individual change in CrCL over time, and *ε*_CrCL,*ij*_ represents the residual error distributed *N*(0, *σ*_CrCL_).

Subsequently, using the observed CrCL values in the current study, we estimated individual empirical Bayes estimates describing the individual predicted changes in CrCL during gestation.

#### 2.4.2. Semiphysiological Model

The individual predicted changes in CrCL derived from the mixed effect model for CrCL were related to the cefazolin CL as follows:

(6) $$\begin{array}{c} {\text{CL}}_{i}=\theta_{\text{CL}0}+\theta_{\text{CL}\;\text{preg}}\times \left(\frac{{\text{CrCL}}_{i}\left(t\right)}{{{\text{CrCL}}_{i}}_{0}}\right), \end{array}$$

where CL_*i*_ is the individual estimate for CL, *θ*_CL0_ represents non-CrCL-related clearance, and *θ*_CL preg_ represents GFR-related clearance, which is multiplied by the normalized change in CrCL, obtained by the ratio between the individual predicted CrCL_*i*_ at time *t* and the baseline CrCL_*i*0_ prior to start of pregnancy. Finally, we also evaluated if additional gestational effects could be identified after inclusion of the predicted change in CrCL.

### 2.5. Handling of Missing Data

For some patients, demographic values were missing. For 46% of patients, body weight was not available. For 55% of patients, age was not available. These were imputed by taking the median age and weight from the pooled dataset. In 58% of patients, not all serum creatinine values were available. These were either imputed based on the median value, or in case of the semiphysiological approach, associated CrCL values were imputed by the typical change in CrCL.

### 2.6. Simulations

Stochastic simulations (*n* = 1000) of dose regimens were performed to compare the predicted concentration-time profiles for the empirical and the semiphysiological model. We performed simulations for different periods of pregnancy. We assumed infection with coagulase negative *Staphylococcus* [9], which has a reported 90% minimum inhibitory concentration (MIC90) between 0.5 and 4 mg/L. Simulations were first conducted using the default dosing regimen of 2 g every 8 hours infused over 30 minutes, which was the general practice dosing regimen used in this study, applying both modelling approaches. Subsequently, we evaluated alternative clinically feasible adaptations to current dosing guidelines used that result in improved time above a MIC90 of 4 mg/L. For this simulation exercise it was explicitly not our aim to derive definitive dose regimens for this infection, but only to provide a motivating proof of concept example.

### 2.7. Software and Estimation Methods

Model development and simulation studies were performed with the software package NONMEM version 7.1 [16] using the first-order conditional estimation method with *η*-*σ* interaction.

### 2.8. Model Selection and Evaluation

Model development of the base and covariate models was guided by the change in objective function value (OFV), plausible parameter estimates, adequate parameter precision, and inspection of goodness-of-fit plots. For development of the base model and the inclusion of covariates in the empirical model, a statistical significance criterion of *P* < 0.05 (decrease in OFV > 3.84) was used. Final models were evaluated using a visual predictive check (VPC) and goodness-of-fit plots. We did not use bootstrapping because of the diversity of subjects in the dataset (e.g., heterogeneous and sparse data) and the time-varying nature of the gestational effect, which would result in bootstrap datasets not representative for the original dataset.

## 3. Results

### 3.1. Base Pharmacokinetic Model

A two-compartmental model best described the data, in line with previous publication [9]. Nonlinear protein-binding models could not be identified based on the available data and were visually not observable. The relationship between free and total cefazolin concentrations was best described using a constant binding model as follows:

(7) $$\begin{array}{c} C_{\text{total}}=\frac{C_{\text{free}}}{f_{u}}, \end{array}$$

where *C*_total_ represents the total drug concentration and *f*_*u*_ represents the (estimated) fraction of free drug. The parameter estimates of the base structural model without gestational effects are provided in Table 2. All fixed effect parameters were estimated with good precision (RSE < 25%).

### 3.2. Empirical Gestational Effect Model

During model building of the empirical model only a linear relationship between CL and GA was found to be significant (dOFV = −9.6, *P* < 0.0025). Relating CL to CrCL did not result in a statistically significant change (dOFV = −0.9) with a highly imprecise covariate slope estimate (RSE 130%), indicating that the available range of renal function did not allow estimation of this (expected) covariate relationship.

Finally, relationships between GA and the central and peripheral volumes of distribution and between free and albumin levels were not statistically significant. Power-type covariate relationships did not describe the observed data better compared to the linear slope-intercept models.

**Figure 1:**
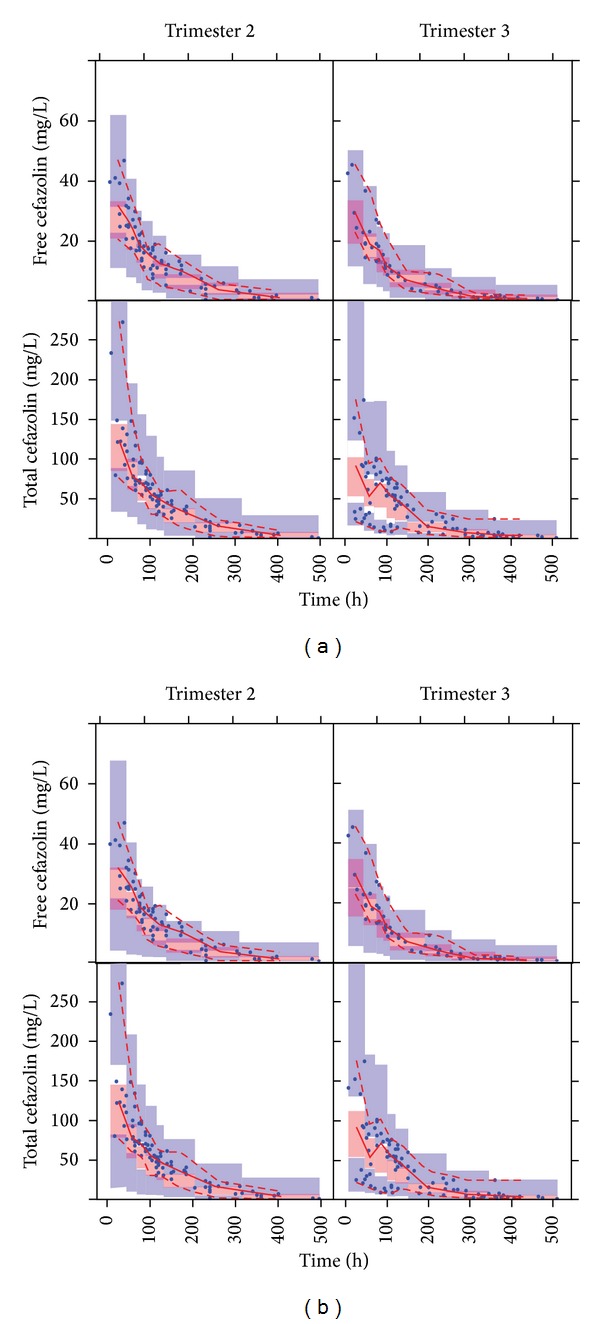
Simulated (areas) and observed (solid circles, lines) free and total cefazolin concentrations versus time as predicted by the empirical model (a) and semiphysiological model (b) for trimesters 2 and 3 of pregnancy. Simulations are represented as the parametric 95% confidence intervals of the simulated 50th (red), 5th, and 95th (blue) percentiles. For the observed values, the 50th (continuous line), 5th, and 95th (dashed lines) percentiles are depicted in red.

**Figure 2:**
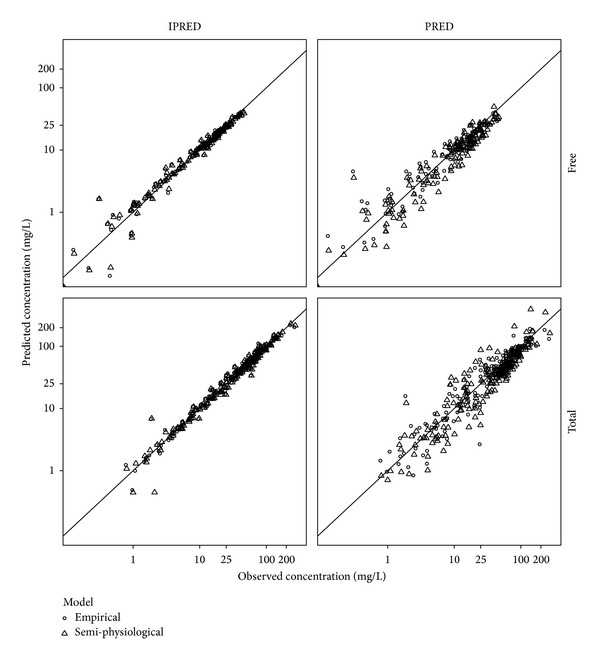
Observed versus individual (IPRED) and population (PRED) predicted free and total cefazolin concentrations (mg/L), for both the empirical approach (open circles) and the semiphysiological approach (open triangles).

The parameter estimates of final covariate model with a linear relationship between CL and GA are provided in Table 2. The precision of the fixed effect parameters was good (RSE < 27%) except for clearance (RSE 58%). We consider this to be related to the simultaneous estimation of two clearance components based on the limited amount of data available. The visual predictive (VPC) check describes (Figure 1(a)) the percentiles (5th, 50th, and 95th) of the model simulated concentration-time curves based on the original study design with the areas representing the 95% confidence intervals around the simulation percentiles. The observed data is included as solid points together with the associated percentiles. Based on this figure we concluded that the typical time course and variability were adequately described, because the simulated and observed percentiles were in good agreement with each other. No trends were observed in the goodness-of-fit plots depicting the observed versus the individual and population predicted free and total cefazolin concentrations (Figure 2).

### 3.3. Semiphysiological Gestational Effect Model

#### 3.3.1. Creatinine Clearance Mixed Effect Model

**Figure 3:**
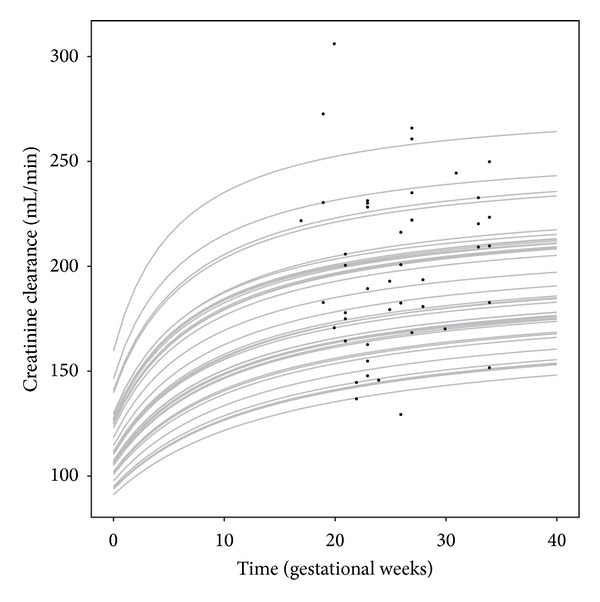
Individual predictions (gray lines) and observed values (solid circles) for the change in CrCL (mL/min) versus time in gestational weeks as predicted by the developed mixed effect model for creatinine clearance.

The parameter estimates for the mixed effect model for CrCL are provided in Table 3. Here, only the random effect parameters were estimated, whereas population parameter estimates were based on previously estimated values in a meta-analysis of the literature report changes in CrCL during pregnancy [13]. Estimates for baseline CrCL and the maximum CrCL were estimated at moderate magnitudes <35.1 CV% with good precision (RSE < 17%). The magnitude of IIV for half-maximum CrCL was high (111.8 CV%) but was also associated with a high RSE (121%). Nonetheless we chose to retain this random effect as it provided a substantially improved description of the CrCL observations, which was the primary aim of this model. An illustration of the individual predicted CrCL dynamics for the available CrCL observations is provided in Figure 3. These predictions are driven by the variability observed for the observations combined with previously established dynamical changes in CrCL during pregnancy. This figure is merely intended as an illustration of how predictions of CrCL were generated and specifically not as a goodness-of-fit plot.

#### 3.3.2. Semiphysiological PK Model

Subsequently, we included the individual predicted changes in CrCL in the base PK model. Estimation of additional effects of GA on PK parameters (i.e., not related to change in GFR) did not further improve model fit, indicating that the pregnancy-related effects could be fully explained by inclusion of the CrCL mixed effect model predictions. The parameter estimates of the resulting model are provided in Table 2. Similar to the empirical covariate model the precision of clearance was relatively high (RSE 44%), whereas other fixed effects had better precision, which was considered to be related to the sparseness of the data. The visual predicted check (Figure 1(b)) and goodness-of-fit plots (Figure 2) indicated adequate description of the data, which were also in agreement with the predictions generated by the empirical PK model.

#### 3.3.3. Simulations

**Figure 4:**
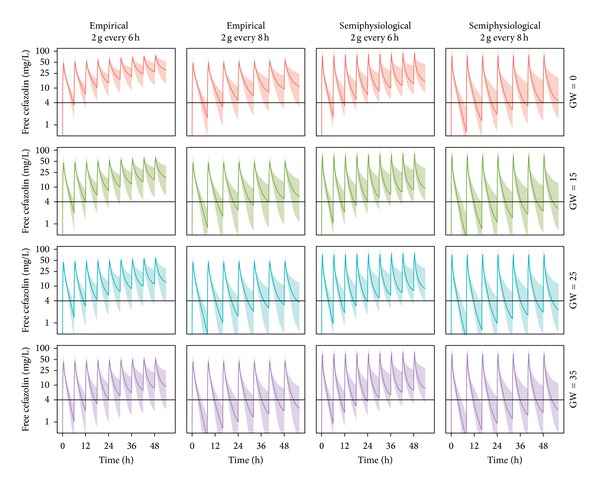
Simulations of typical free and total cefazolin concentration-time profiles using the empirical and the semiphysiological approach at different periods of pregnancy (gestational weeks) based on a dose regimen of 2 g cefazolin every 6 or 8 hours administered over 30 minutes. The expected 90% minimum inhibitory concentration is depicted as horizontal black line. GW: gestational weeks.

The empirical and semiphysiological models were used for simulations to assess the differences in model prediction across the full duration of gestation (Figure 4). When comparing the magnitude of change in concentration-time profiles across gestation, a difference was observed between the empirical and semiphysiological approaches, especially in early pregnancy. As the semiphysiological model is based on the expected time course of renal function across pregnancy, we may assume that these predictions will be closer to the true change in cefazolin PK, compared to the linearly interpolated change for the empirical model. By simulation of alternative dose regimens we demonstrated that an increased dosing interval of 2 g cefazolin every 6 hours (instead of 8 hours) yielded prolonged therapeutic concentrations, based on the expected MIC90 value of 4 mg/L, yet remained feasible for implementation in daily clinical practice.

## 4. Discussion 

This work described the first population PK model describing free and total cefazolin in pregnant patients, which can be used to rationally inform informative dose regimens in pregnant patients using a model-based approach [17, 18]. In addition we demonstrated a novel hybrid approach of leveraging prior knowledge on physiological changes in a population PK model, which may help to derive more informative and potentially more clinically relevant models in situations with sparse data.

Although both modeling approaches showed comparable description of the observed data, a number of advantages of the semiphysiological approach can be recognized. The semiphysiological model included a more realistic change in CrCL compared to the linear pregnancy effect in the empirical model. The difference between these two strategies becomes clear when extrapolating outside the observed range of GAs (17–40 weeks). For the purpose of extrapolation, we expect that the semiphysiological approach may be closer to the true change in clearance. As pregnancy is not a dichotomous variable, the time-varying change should be considered for potential dose regimen adjustments. However, when PK studies aiming to derive dose regimens only recruit patients in specific parts of pregnancy, required dose regimens in other periods of pregnancy may still be different. When appropriate PK data is missing, the proposed modeling approach may be considered. Secondly the semiphysiological modeling approach may also be of relevance to generate informative PK study designs in pregnant patients based on available knowledge, as has been demonstrated previously [13].

Although it is known that CrCL will be a key covariate for prediction of cefazolin clearance, we did not find such an effect for the empirical model, whereas the gestational age, with no missing data, was a strong covariate in the empirical model. We consider this finding to be related to the relatively high number of missing CrCL values and the crude method for imputation of such values. As such the GA resulted in a much more informative covariate. In contrast, for the semiphysiological approach the impact of CrCL could be incorporated and resulted in a model with a comparable description of the data.

The predictions of the semiphysiological model are driven by the previously developed structural model describing the mean gestational dynamics of CrCL. This previous analysis had some limitations as it was based on relatively old studies with potentially different methods [14]. Nonetheless, this analysis describes in a reasonably good way what is known about changes in CrCL. Although the predicted changes in CrCL are inherently associated with some uncertainty, the mean population predictions did not show any trend indicating limited impact of any bias in CrCL predictions that may be present.

Age and weight values used for the computation of CrCL were missing for a substantial number of patients, which were imputed based on median values. This imputation approach can result in some shrinkage of the individual CrCL values towards the population mean, which may lead to potentially biased effects of CrCL on CL. The impact of missing age values might be limited as pregnant females can be expected to reside in a relatively narrow age range. During model building we observed the potential impact of CrCL imputation, as GA was a far superior covariate compared to CrCL in contrast to what was expected. The semiphysiological model on the other hand was able to derive a comparable description of the data using CrCL values, potentially because the CrCL mixed effect model improves the imputation of gestational changes in CrCL. An alternative and potentially improved imputation approach would be joint modeling of CrCL based on GA (which is available in all patients). Ultimately we did not evaluate a joint modeling approach as it was considered potentially challenging to develop such a model based on the limited data available and it would still remain doubtful to what extent this imputation method would lead to a decrease in bias. We, however, expect that regardless of the choice of imputation method this remained a sparse dataset with substantial limitations which should be acknowledged and carefully considered when applying this model for future simulation purposes.

Studies in pregnant patients often have substantial design limitations, as was also the case in the current analysis. This leads to limitations in any inference to be derived but likewise also demonstrates the importance of using efficient analysis strategies that utilize prior knowledge when available. In our case the developed semiphysiological model for cefazolin showed comparable description of the observed data, yet it allowed for more realistic extrapolation of expected cefazolin concentrations outside the range of gestational ages available.

This semiphysiological approach can be considered for leveraging known physiological changes from the literature into a population PK analysis in order to support and inform PK models that are based on a limited amount of data, such as is frequently the case for PK studies during pregnancy. This approach might also be applicable to other drugs in situations involving sparse data situations involving previously established and relevant physiological knowledge. However ultimately, the conduct of well-designed prospective PK studies will remain the golden standard to evaluate PK and optimize dose regimens.

## Figures and Tables

**Table 1:** Patient demographics and study characteristics.

Characteristic	Value
Number of patients	94
Gestational age (weeks) (median, range)	33 (17–40)
Age (years) (median, range)	31 (20–42)
Body weight (kg) (median, range)	72 (54–99)
Serum creatinine (mg/dL) (median, range)	0.64 (0.33–0.88)

**Table 2:** Parameter estimates of the base and final population PK models (empirical and semiphysiological) for cefazolin.

Description	Parameter	Unit	Estimates (RSE%)		
			Base model	Empirical model CL~GA^a^	Semiphysiological Model CL~CrCL^b^
Structural model					
Clearance	*θ*_CL0_	L/min	0.49 (7)	0.119 (58)	0.142 (44)
Central volume	*V*_*C*_	L	32.5 (16)	33.1 (17)	14.1 (25)
Peripheral volume	*V*_*P*_	L	12.8 (25)	12.8 (27)	17.1 (7)
Intercompartmental clearance	*Q*	L/min	0.335 (21)	0.326 (25)	0.436 (10)
Free fraction	*F*_*U*_	—	0.289 (5)	0.286 (5)	0.291 (9)
Gestation effect on clearance	*θ*_CL Preg_		—	0.217 (16)	0.212 (38)
Between subject variability					
Clearance	*ω*_CL_	CV%	22.1 (25)	19.9 (24)	10.4 (70)
Central volume	*ω*_*V*1_	CV%	49.1 (22)	47.6 (21)	101.5 (21)
Peripheral volume	*ω*_*V*2_	CV%	32.7 (41)	34.2 (41)	68 (26)
Free fraction	*ω*_*F*_*U*__	CV%	18.9 (36)	17.5 (37)	19.1 (38)
Residual unexplained variability variances					
Proportional, free concentration	*σ*_FP_		0.0162 (8)	0.0158 (11)	0.0153 (8)
Additive, free concentration	*σ*_FA_		0.176 (34)	0.229 (42)	0.217 (36)
Proportional, total concentration	*σ*_TP_		0.0348 (8)	0.0328 (10)	0.0328 (9)
Additive, total concentration	*σ*_TA_		0.662 (48)	0.842 (46)	0.681 (25)

RSE: relative standard error; GA: gestational age (weeks). ^a^Empirical model: CL = *θ*_CL0_ + *θ*_CL Preg_∗(CrCL_*i*_(*t*)/CRCL_*i*0_); ^b^semiphysiological model: CL = *θ*_CL0_ + *θ*_CL Preg_∗(1 + (GA/40)).

**Table 3:** Parameter estimates of the nonlinear mixed effect model for change in creatinine clearance (CrCL).

Description	Parameter	Unit	Estimates (RSE%)
Structural model estimates as reported in a previous meta-analysis (13)			
Baseline CrCL	CrCL_0_	mL/min	97.83 (3.91)*
Maximum CrCL	CrCL_MAX_	—	83.83 (12.48)*
Time of half-maximum CrCL	CrCL_50_	weeks	13.3 (37.59)*

Between subject variability (CV%)			
Baseline CrCL	*ω*_CrCL_0__	CV%	31.4 (16)
Maximum CrCL	*ω*_CrCL_MAX__	CV%	35.1 (17)
Time of half-maximum CrCL	*ω*_CrCL_50__	CV%	111.8 (121)

Residual unexplained variability variance			
Proportional error	*σ*_CrCL_		0.0291 (43)

CrCL: creatinine clearance; RSE: relative standard error. *These values were fixed during estimation of the mixed effect model; that is, only random effects were estimated.
